# Leukocyte Nucleolus and *Anisakis pegreffii*—When Falling Apart Means Falling in Place

**DOI:** 10.3390/genes11060688

**Published:** 2020-06-23

**Authors:** Ivona Mladineo, Jerko Hrabar

**Affiliations:** Institute for Oceanography and Fisheries, 21000 Split, Croatia; hrabar@izor.hr

**Keywords:** *Anisakis pegreffii*, nucleolus, nucleolar stress, peripheral blood mononuclear leukocytes, Sprague Dawley rat

## Abstract

The view of the nucleolus as a mere ribosomal factory has been recently expanded, highlighting its essential role in immune and stress-related signalling and orchestrating. It has been shown that the nucleolus structure, formed around nucleolus organiser regions (NORs) and attributed Cajal bodies, is prone to disassembly and reassembly correlated to various physiological and pathological stimuli. To evaluate the effect of parasite stimulus on the structure of the leukocyte nucleolus, we exposed rat peripheral blood mononuclear cells (PBMC) to the crude extract of the nematode *A. pegreffii* (Anisakidae), and compared the observed changes to the effect of control (RPMI-1640 media), immunosuppressive (MPA) and immunostimulant treatment (bacterial lipopolysaccharide (LPS) and viral analogue polyinosinic:polycytidylic acid (poly I:C)) by confocal microscopy. Poly I:C triggered the most accentuated changes such as nucleolar fragmentation and structural unravelling, LPS induced nucleolus thickening reminiscent of cell activation, while MPA induced disassembly of dense fibrillar and granular components. *A. pegreffii* crude extract triggered nucleolar segregation, expectedly more enhanced in treatment with a higher dose. This is the first evidence that leukocyte nucleoli already undergo structural changes 12 h post-parasitic stimuli, although these are likely to subside after successful cell activation.

## 1. Introduction

Efficient ribosynthesis is tightly coupled to cell growth, therefore a high transcription rate of rDNA genes and the activity of ribosomal polymerases is essential for rapid cell proliferation required to meet the cellular needs of ribosomes. Under the stress conditions affecting the cell cycle and intracellular energy status (e.g., lack of nutrients), change in ribosynthesis is one of the cell strategies to retrieve homeostasis [1]. Moreover, increased ribosynthesis, apart from being implicated in carcinogenesis [2], is also essential during host immune responses, as it enables its efficient signalling and orchestrating [3,4,5].

Nucleolus structure, formed around nucleolus organiser regions (NORs) in the nucleus, is a result of the processes connected with transcription and processing of precursor ribosomal RNA (pre-rRNA) and assembly of precursors of the small and large ribosome subunits. Accompanied by masses of condensed chromatin, the nucleolus is composed of three different components: the dense fibrillar component (DFC), the granular component (GC), and the fibrillar centre (FC) [6]. The latter is assumed to be a protein storage site, appearing as a distinct spherical body encircled by the DFC and GC [7]. Both compartments contain growing preribosomal particles, the DFC at early, while the GC at late states of formation. Additionally, the nucleolus is in close association with Cajal bodies (CB), which coordinate maturation of specific nuclear RNAs (small nuclear RNA: snRNA; small nucleolar RNA: snoRNA) and histone mRNA processing [8] and likely have a role in the transduction of proliferative signals to the nucleus [2]. Given the close relationship between the nucleolus and the CB, the latter has also been implicated in coordination of the stress response. The CB structural component frequently used as a signature marker is p80-coilin protein. Although found in direct contact with the nucleolus, CBs can travel toward and away from nucleoli, and are possibly engaged in the transfer of the material between the CBs and the nucleoli [9].

The nucleolar architecture depends on the functional state of cells and changes concomitantly with cellular changes. Different types of cellular stress reflect on ribosome subunit production and cell growth, accompanied by dramatic changes in the organisation and composition of the nucleolus [2].

*Anisakis* spp. larvae that accidentally infect humans can elicit four main illness types: gastric, intestinal, ectopic and gastro-allergic [10] that express mostly mild, seldomly severe symptomatology [11]. In infected humans, third-stage larvae (L3) are either unable to develop into pre-adult forth-stage larvae (L4) and reproductively active adults, or the former occurs extremely rarely. This consequently leads to the death and decay of larvae within human tissues, accompanied by a strong host reaction in form of granulomatous inflammation.

The first molecular approach to *A. pegreffii* (Nematoda, Anisakidae) pathogenesis in humans has been documented through in vivo experimental infection of a Sprague Dawley rat [12,13] and in vitro in human fibroblast [14] and dendritic cell lines [15]. Although a typical proinflammatory pathway was inferred in all three models, there were marked differences observed between in vivo and in vitro models, mainly related to the presence of the IL17 signalling pathway exclusively in the former. However, since activation of the IL17 axis has been related to bacterial and fungal infections [16], it remains elusive whether the pathway was induced specifically by *A. pegreffii* infective larvae or/and whether it was triggered by microbiota spreading through rat tissues damaged by the migrating larvae [12]. Although the most marked rat response expectedly consisted of the proinflammatory activation, in vivo *A. pegreffii* infection also triggered a KEGG 03010 ribosome pathway, particularly enriched in rat stomach tissues [12]. While the authors observed no large expression differences of the specific ribosomal genes, these were found to be consistently upregulated across the stomach dataset, which suggested that this perturbation potentially reflects the onset of ribosomal or nucleolar stress [2] taking place in the rat stomach during the larval penetration.

To assess whether the observed ribosome pathway in infected rat stomach tissues [12] is a consequence of nucleolar stress, we exposed rat peripheral blood mononuclear cells (PBMC) to *A. pegreffii* L3 crude extract (CE) and assessed the stability of nuclear and nucleolar proteins engaged in ribosomal synthesis by immunocytochemistry.

## 2. Materials and Methods

### 2.1. Animal Ethics

The Ethical Committee of the School of Medicine at the University of Split (registry number 2181-198-03-04-18-004) and the Veterinary and Food Safety Office of the Ministry of Agriculture (registry number 525-10/0255-16-7) approved all animal experiments and protocols. Rat experiments were performed at the University of Split Animal Facility (permit number HR-POK-019) where they were raised and housed in pairs, in plastic cages with sawdust and corn bedding. The animals were kept in a controlled environment: food and water ad libitum, temperature 22 ± 1 °C, with a 12 h light/dark cycle. The animals were separated in individual cages 24 h prior to the experiment.

### 2.2. *A. pegreffii* Crude Extract (CE)

*A. pegreffii* larvae were collected from the blue whiting *Micromesistius poutassou*, freshly caught in the C1 fishing zone of the Adriatic Sea (FAO 37.2.1) and provided by a trusted dealer. Briefly, actively moving larvae were washed several times in physiological saline solution and checked under an Olympus BX 40 light microscope (Olympus Corp., Shinjuku, Tokyo, Japan) to confirm type I larvae identity. Ca. 400 larvae were washed in PBS (pH 7.4) and deep-frozen for 15 min at −80 °C. Larvae were thawed, dried on paper, weighted so to obtain 0.25 g of larvae/1 mL PBS, frozen again and manually homogenised. Afterwards, the homogenate was sonicated on ice for 60 s (10% duty cycles, 20% power) and centrifuged (600 g/10 s, at 4 °C). The supernatant was collected and filtered through 0.45 mm filters. Concentration was calculated according to Bradford using standard dilutions of bovine serum albumin, resulting in 4 mg/mL from ca. 400 larvae.

### 2.3. Peripheral Blood Mononuclear Cells (PBMC) Immunocytochemistry (ICC) and Confocal Microscopy

Blood was collected by 5 mL syringe-withdrawal from the caudal vein of 6 adult male rats (average weight 197 ± 13.6 g). Prior to cell isolation, a single spherical cover glass, previously coated with cell and tissue adhesive Corning^®^ Cell-Tak (Sigma Aldrich, St. Louis, MO, USA) on the upper surface and left to dry in the microbiological hood, was placed in each well of 24-well plates. For the peripheral blood mononuclear cells (PBMC) isolation, Ficoll-Paque (GE Healthcare Bio-Sciences AB, Uppsala, Sweden) protocol was followed that enabled isolation of 2 × 10^6^ leukocytes in RPMI-1640 supplemented with 10 units/mL of heparin, 2% foetal calf serum (FCS) (Invitrogen Life Technologies, Carlsbad, CA, USA) and 1% penicillin/streptomycin (10,000 U/mL Penicillin G sodium; 10,000 mg/mL Streptomycin sulphate). Cells were then seeded in 24-well plates with spherical cover glasses, and left to settle for 1 h in an incubator at 36 °C and 5% CO_2_ before stimulation by: (i) 5 µg *A. pegreffii* crude extract (CE; A5); (ii) 0.5 µg *A. pegreffii* CE (A0.5); (iii) LPS (*Escherichia coli* 0111:B4 lipopolysaccharides); (iv) polyinosinic:polycytidylic acid sodium salt (poly I:C) that induces interferon-γ (IFN-γ)-mediated response; (v) mycophenolic acid (MPA), an effective immunosuppressive drug and metabolite of mycophenolate mofetil [17] and (vi) RPMI-1640 (as negative control), each in quadruplicate (Table 1). Another 24-well plate was used with the same experimental setup, but the secondary antibodies were omitted during the procedure.

Twelve h post-stimulation the spherical cover glasses with adhered cells were collected, washed in 0.1% Tween 20 in PBS (PBST), fixed in cold methanol for 5 min, permeabilised by 0.25% Triton X and 1% foetal bovine serum (FBS) in PBS for 10 min and washed three times in PBS. Background fluorescence was blocked by adding a few drops of Image-iT^®^ FX signal enhancer following the producer’s manual (ThermoFisher Scientific, Waltham, MA, USA). Primary antibodies (all from Abcam, Cambridge, UK): rabbit monoclonal anti-Nop58 (targeting DFC; 1:250, ab155969), AlexaFluor 647 conjugated mouse monoclonal anti-coilin (targeting CB; 1:100, ab196714) and mouse monoclonal anti-nucleophosmin (B23/NPM1; targeting GC; 1:500, ab10530), were added together in 1% BSA in PBST and incubated on the cell layer for 1 h at room temperature. After the washing steps in PBS, secondary antibodies; donkey F(ab’)2 anti-rabbit IgG H&L AlexaFluor 568 (1:200, ab175694) and goat anti-mouse IgG AlexaFluor 488 (1:200, ab150117) added in 1% BSA in PBST, were incubated onto cells for 1 h at room temperature. Following washing, cells were mounted in FluoroShield with 4,6-diamidino-2-phenylindole (DAPI) (Sigma Aldrich, St. Louis, MO, USA) for DNA labelling. Slide-mounted cells were observed with a TCS SP8 X confocal microscope (Leica, Wetzlar, Germany) at Institute Rudjer Boskovic, Zagreb.

## 3. Results

**Figure 1:**
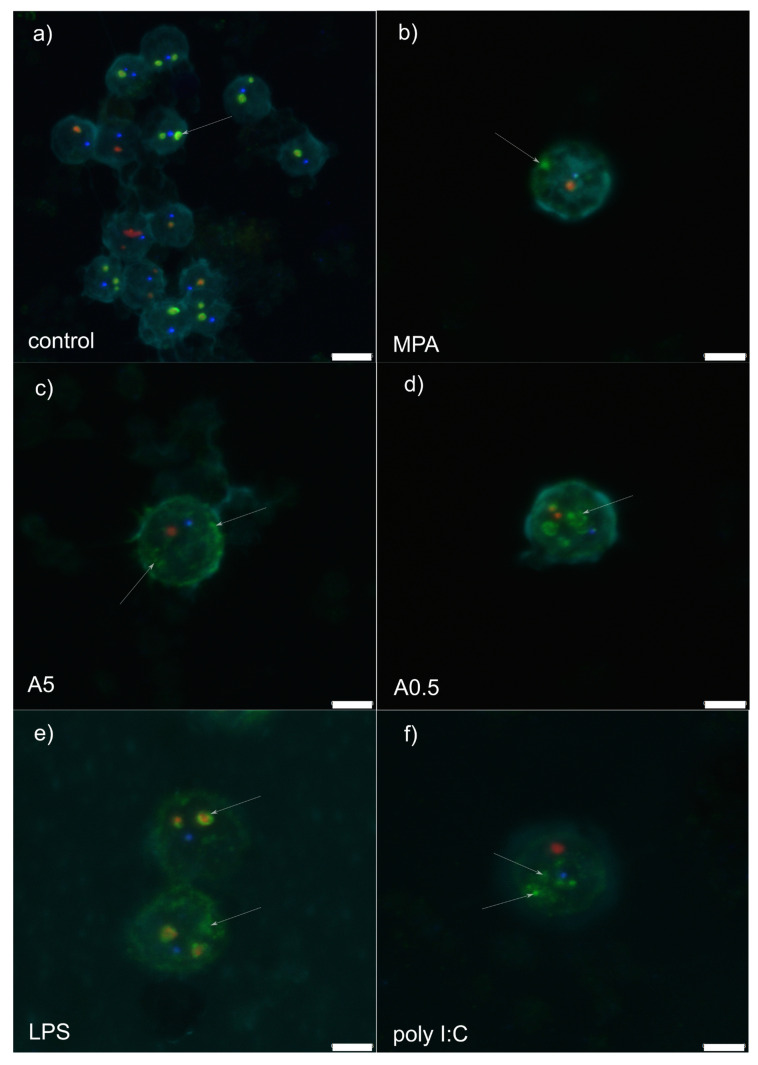
Z-stack representative microphotograph of immunocytochemical localization of Cajal bodies (coilin, in blue), granular components (B23/NPM1, in green), dense fibrillar components (Nop58; in red) and cell nucleus (pale blue, labelled by 4,6-diamidino-2-phenylindole [DAPI]) in rat peripheral blood mononuclear cells 12 h post-stimulation with: (**a**) RPMI-1640 medium (control); (**b**) mycophenolic acid (MPA; immunosuppressor); (**c**) 5 µg of *A. pegreffii* crude extract; (**d**) 0.5 µg of *A. pegreffii* crude extract; (**e**) bacterial lipopolysaccharide (LPS); (**f**) polyinosinic:polycytidylic acid (poly I:C; viral analogue). White arrows point to the appearance of the granular components. Scale bars: 5 µm (**a**), 3 µm (**b**–**f**).

**Figure 2:**
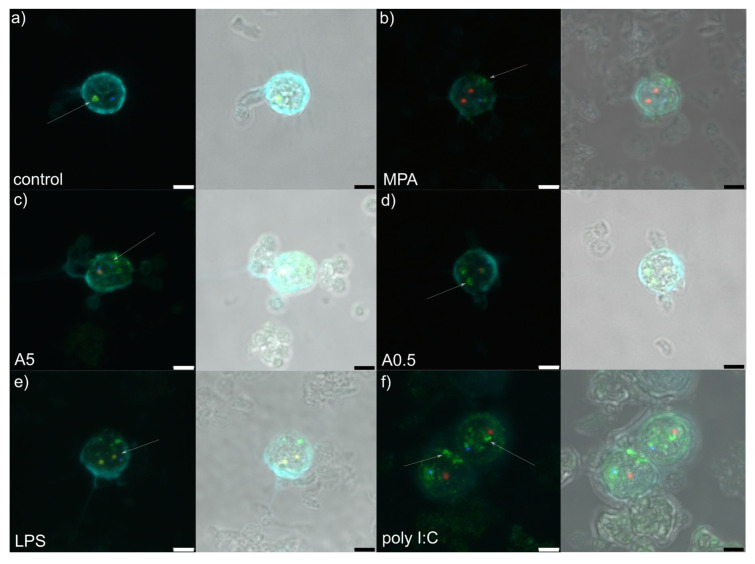
Z-stack representative microphotograph of immunocytochemical localization of Cajal bodies (coilin, in blue), granular components (B23/NPM1, in green), dense fibrillar components (Nop58; in red) and cell nucleus (pale blue, labelled by 4,6-diamidino-2-phenylindole [DAPI]) in rat peripheral blood mononuclear cells 12 h post-stimulation with: (**a**) RPMI-1640 medium (control); (**b**) mycophenolic acid (MPA; immunosuppressor); (**c**) 5 µg of *A. pegreffii* crude extract; (**d**) 0.5 µg of *A. pegreffii* crude extract; (**e**) bacterial lipopolysaccharide (LPS); (**f**) polyinosinic:polycytidylic acid (poly I:C; viral analogue). Signals are also viewed overlaid in a bright field microphotograph. White arrows point to the appearance of the granular components. Scale bars: 3 µm.

RPMI stimulation (control treatment) showed granular component (GC: labelled by nucleophosmin in green) forming a compact and regular ring enveloping small dense fibrillar components (DFCs) labelled by NOP58 (red) (Figure 1a and Figure 2a). Mostly 1–3 such structures were observed per nucleolus. Small, regular and spherical Cajal bodies (CBs: 1–2 per nucleolus) labelled by coilin (blue) were observed in close proximity to the GCs.

Mycophenolic acid stimulation (immunosuppressor, negative control) induced dissociation of the GC from DFC (Figure 1b and Figure 2b). The structure of the former was less compact than in RPMI-1640-stimulated cells, showing fuzzy contours and occasional nucleophosmin speckles dispersed throughout the nucleus. In general, the GC was observed at the nucleus periphery. A single or two small, spot-like DFCs were usually observed, accompanied by a single translucent CB.

Stimulation by 5 µg of *A. pegreffii* CE induced strong dispersion of nucleophosmin throughout the nucleus and its accumulation at the nuclear periphery in the form of multiple granular fragments (Figure 1c and Figure 2c). Usually 1–2 weakly labelled and enlarged DFCs were observed surrounded only by a thin ring of nucleophosmin. Compact CB was not observed in close proximity to the DFC as a rule.

Stimulation by 0.5 µg of *A. pegreffii* CE induced development of multiple, large and dense, mulberry-like GCs that prevailed in numbers compared to the DFCs and were located centrally in the nucleus (Figure 1d and Figure 2d). Sometimes nucleophosmin granular speckles filled the whole nuclear space. DFCs were small and present in pairs with or without a compact nucleophosmin ring. One to two CBs were found displaced from the DFC.

Stimulation by bacterial lipopolysaccharide (LPS; mimicking bacterial infection) triggered similar changes in the architecture as 0.5 µg of *A. pegreffii*, with the difference that GC was, by rule, found enveloping enlarged DFCs and also scattered in granulated form throughout the nucleus (Figure 1e and Figure 2e). CBs were observed between two DFCs.

Polyinosinic:polycytidylic acid (Poly I:C; analogue of viral infection) induced detachment of the DFC and GC (Figure 1f and Figure 2f). The former was usually singular and enlarged, while the latter was small, compact and granulated, dispersed from the centre up to nuclear periphery (“milky-way appearance”). A single CB was also displaced from the DFC.

Without use of markers to differentiate between cell types within PBMCs that in humans mainly consist of lymphocytes (70–90%), of which 45–70% are CD3+ T cells, and to a lesser degree of monocytes (10–30%), dendritic cells (1–2%) and stem cells (0.1–0.2%), we can estimate that the observed changes were mainly described in T cells.

## 4. Discussion

During the physiological cell cycle in higher eukaryotes, ribosome production starts at the end of mitosis, increases during G1, is maximal in G2 and stops during prophase, at the end of which dissociation of the nucleophosmin proteins from the nucleolus occurs [18], changing the nucleolar shape and forming a 3-D network [19]. At the end of prophase, rDNA transcription is terminated, the nucleolar processing proteins from the DFC and GC are dispersed around condensing chromosomes and the nuclear envelope is disrupted, depicting the state of nucleolar disassembly [19]. To follow at the onset of telophase, an early and complex event of nucleolar assembly takes place over a relatively long period compared to nucleolar disassembly. Under stress conditions, the nucleolus shows alterations in its structural appearance and function, which have been described under the term nucleolar stress. Since such alterations consequently impair homeostasis of the ribosynthesis and activate cellular stress response through p53 or other stress signalling pathways, the process has been also referred as ribosomal or ribotoxic stress [20]. Here we inferred that stimulated leukocytes during the early phase of antigen presentation (12 h post-stimulation) undergo marked nucleolar morphological alterations reminiscent to that observed during the nucleolar stress. This has been previously corroborated only at the transcriptomic level through an increased expression of ribosomal proteins that suggested a state of ribosomal stress [12]. Therefore, our preliminary data suggest that in the case of mononuclear immune cells, structural changes of the nucleolus as a response to antigen stimuli are essential to evoke the processes necessary for normal activation and proliferation of targeted cells. Unfortunately, the study design did not allow sampling of later time points to evaluate when the nucleolus re-assembles once the antigen is processed or removed from the cell environment.

Observed nucleolar alterations have been the least substantial in MPA-stimulated cells. Mycophenolic acid (MPA) is a well-known guanine inhibitor acting as an immunosuppressive agent that blocks ribosomal (rRNA) synthesis and T cell proliferation [17]. Guanine, along with other purine and pyrimidine nucleotides, plays critical roles in the development, activation and survival of mature T lymphocytes, while its inhibition of synthesis by MPA results in T cells apoptosis. However, the proapoptotic activity is evident only in cycling and not in merely activated T lymphocytes [21]. Purines control both G1 to S phase transition and progression through the S phase, so when inhibited at the onset of T cell activation, lymphocytes are arrested in the G1 phase with a completely abrogated expression of cyclins and cyclin-dependent kinases (CDKs). In contrast, when MPA inhibits purines in already activated and cycling T cells, cells enter the S phase but their progression from early to intermediate S phase is blocked. Exposing naive T cells to MPA herein, suggests that observed nucleolar changes could be antecedent of the upcoming apoptosis. In addition, observed nucleolar disassembly was mild in respect to other stimuli, which potentially could be related to the MPA’s inhibition of CDKs. Namely, only high levels of Cdk1 activity enable the nucleolar disassembly in the prophase [22], while MPA suppression maintains some nucleolus integrity, but consequently shifts the cell towards apoptosis [21].

Disassembly of the nucleolus after application of two *A. pegreffii* crude extract concentrations (i.e., 0.5 and 5 µg) showed remarkable differences in the range of structural changes, the latter expectedly being more enhanced at higher concentration. It resembles more a nucleolar segregation rather than fragmentation, the former characterised by condensation and subsequent separation of the FC and GC, together with the formation of “nucleolar caps” around the nucleolar remnants [23]. This is usually triggered by DNA damage and/or transcriptional inhibition [24,25], while nucleolar caps are formed by nucleolar proteins such as UBF (nucleolar transcription factor 1), nucleoplasmic proteins, mostly RNA-binding proteins and coilin. This is the first evidence that the parasite-derived crude extract induces nucleolar segregation in leukocytes. Interestingly, LPS-stimulated cells showed consistently compact structure of the DFC (marked in red by Nop58) and GC (marked in green by nucleophosmin), but also accompanied by an increase in granulated nucleophosmin scattered throughout the nucleus. In the physiological cell cycle, B23/nucleophosmin (B23/NPM1) constantly shuttles between the nucleus and the cytoplasm [26]. It participates in diverse cellular processes, such as ribosome biogenesis, DNA replication and repair, stress response, centrosome duplication and nucleo-cytoplasmic traffic, as well as interaction with several viral proteins in different phases of the infection [27]. Our data suggest that in 12-h-LPS-stimulated cells, nucleophosmin retains its structure within the GC, but it becomes additionally upregulated to meet the metabolic demand of the stimulated leukocytes. Previous studies evidenced that LPS- and phytohemagglutinin (PHA)-stimulated nucleoli tended to fuse and enlarge in lymphocytes, macrophages and dendritic cells, activating the Toll-like receptor 4 (TLR4) or Toll-like receptor 9 (TLR9) signalling pathway, clearly evidencing the role of the nucleolus in the immune system [28,29,30]. Although we did not infer the physical fusion of the nucleoli, which might be attributed to different microscopy techniques applied, we noticed a considerable “thickening” of the nucleophosmin, suggesting the consistency between our and the aforementioned previous studies.

In contrast, the nucleolar changes observed in cells stimulated by polyinosinic:polycytidylic acid (poly I:C) resemble more a nucleolar fragmentation characterised by the unravelling of the FC. Such alteration has been attributed to cell stress evoked by inhibition of either RNA polymerase II (RNA Pol II) or protein kinases [31,32]. Poly I:C is a synthetic analogue of double-stranded RNA present in some viruses, which interacts with endosomal toll-like receptor 3 (TLR3) on the B lymphocytes and antigen-presenting cells [33] and leads to the production of type I interferons (IFNs) and inflammatory cytokines. We used poly I:C to mimic viral infection in PBMC and assess potential nucleolar changes. In cells infected with various types of viruses, such as adenovirus, coronavirus, hepatitis C virus, human immunodeficiency virus, human papillomavirus, herpes simplex virus type 1, poliovirus and West Nile virus, the nucleolus is prone to profound alterations in structure and composition as it plays a major role in the virus life cycle, independent of their replication site [34]. However, in addition to virus-induced changes in nucleolar morphology and proteome, the most intensive alterations develop in Cajal bodies (CBs) in the form of coilin accumulation, and formation of nucleoplasmic microfoci and rosettes [2]. In our case, whether CBs disruption would follow the observed nucleolus fragmentation and unravelling of components, remains to be tested, as the feature was not detected within 12 h post-stimulation. Worth mentioning is that the nucleolar changes induced by poly I:C were the most dramatic among all assessed stimuli.

Interestingly, we observed no major changes in the number or structure of CBs after stimulation with different stressors, except for a more translucent appearance in respect to unstimulated cells. CBs coordinate maturation and processing of small nuclear RNA (snRNA), small nucleolar RNA (snoRNA) and histone mRNAs, while its hallmark protein coilin (p80-coilin) is particularly altered during viral infections [2]. During DNA damage (UV irradiation, drug inhibition of topoisomerase II) or a transcriptional inhibition (actinomycin D), segregated nucleolus results in development of the aforementioned nucleolar caps consisting of an array of nucleolar proteins, among which we focused herein on coilin. Observing no formation of nucleolar caps in stimulated-rat PBMC, concordant to some other studies (see [2]), we suggest the most robust explanation that the effectuated stressors do not evoke DNA damage nor transcriptional inhibition in cells. We can only speculate whether the translucent appearance of the CBs may indeed indicates coilin depletion in the nucleolus that subsequently would induce its disruption and finally defects in snRNP biogenesis [35].

## Figures and Tables

**Table 1:** Experimental design of the peripheral blood mononuclear cells (PBMC) immunocytochemistry (ICC) protocol developed in 24-well plates and morphological appearance of the cell nucleoli.

	Treatment	DFC	GC	CB
N of wells/treatment		4	4	4
Primary ab		rabbit monoclonal anti-Nop58	mouse monoclonal anti-nucleophosmin	AlexaFluor 647 conjugated mouse monoclonal anti-coilin
Secondary ab		donkey F(ab’)2 anti-rabbit IgG H&L AlexaFluor 568	goat anti-mouse IgG AlexaFluor 488	n/a
Appearance	*A. pegreffii* crude extract (5.0 µg)	weak labelling, enlarged	strong dispersion and peripheral accumulation of multiple granular fragments	compact
	*A. pegreffii* crude extract (0.5 µg)	small, paired, with or without compact GC ring	multiple, large, dense, mulberry-like	displaced from DFC
	LPS	small, compact	enveloping enlarged DFCs and scattered in granula	close to DFC
	poly I:C	single, enlarged	detached from DFC, “milky-way” appearance	single, displaced from DFC
	MPA	small, spot-like	dissociation from DFC, fuzzy speckled	single, translucent
	control (RPMI-1640)	small and compact	compact and regular ring enveloping DFC	small, regular, spherical, proximal to GC

DFC: dense fibrillar component; GC: granular component; CB: Cajal body; LPS: *Escherichia coli* 0111:B4 lipopolysaccharides; poly I:C: polyinosinic:polycytidylic acid sodium salt; MPA: mycophenolic acid.
